# Characterization of phytoconstituents and evaluation of total phenolic content, anthelmintic, and antimicrobial activities of *Solanum violaceum* Ortega 

**Published:** 2013

**Authors:** Golam Sarwar Raju, Mizanur Rahman Moghal, Syed Masudur Rahman Dewan, Mohammad Nurul Amin, Mustahsan Billah

**Affiliations:** 1 *Department of Pharmacy, Noakhali Science and Technology University, Sonapur, Noakhali- 3814, Bangladesh*

**Keywords:** Broad-spectrum, Phytoconstituents, *Solanum violaceum*, Total phenolic content, Vermicidal activity

## Abstract

**Objective:** The present study was conducted to detect possible chemicals (phytoconstituents), prove ethno-medicinal value of the plant, and investigate antimicrobial, anthelmintic, and total phenolic content of crude methanolic extract of the *Solanum violaceum *plant*.*

**Materials and Methods:** Phytochemical screening was carried out using different chemical group test methods. In anthelmintic activity test (using *Pheretima posthuma *model), five concentrations (10, 20, 30, 40, 50, and 80 mg/ml in distilled water) of extracts and albendazole as standard were used which involved the vermifuge and vermicidal activity on the worms. For the evaluation of *in vitro *antimicrobial activity, disc diffusion method, and to determine the total phenolic content, Folin-Ceocalteu method (gallic acid as standard) were used.

**Results:** The phytoconstituent analysis revealed presence of alkaloids, carbohydrate, glycoside, flavonoid, saponin, gum, diterpenes, phenol, protein, and tannin. The crude extract exhibited significant anthelmintic property comparing with the standard. The methanolic extract revealed broad-spectrum antimicrobial activity at the concentration of 400 µg/disc. The results were compared with that of the standard ciprofloxacin. The extract exhibited moderate amount of total phenolic compound (54.67±1.18 mg/gm of gallic acid equivalent).

**Conclusion:** Since *S. violaceum* have shown antimicrobial, antioxidant, and anthelmintic activities, more studies such as anti-inflammatory, analgesic, antipyretic, and other pharmacological activities should be carried out to justify its traditional use, as the plant is available and used broadly in the rural areas for folkloric remedies.

## Introduction

**For** the development of health in mankind, the medicinal plants always play an important role. According to World Health Organization, more than 80% of world’s population is reliant on medicinal plants to maintain their health and to cure their ailments (Alves and Rosa, 2005 ▶). It is now established and fully believed that phytoconstituents obtained from the medicinal plants serve as pilot molecules in the modern medicines (Ncube et al., 2008 ▶) and many people still depend on the traditional medicine for their preliminary health care and treatment (Bannerman et al., 1986 ▶).

There are about 90 genera and between 2000 to 3000 species of Solanaceae which are widely spread throughout tropical and temperate regions of the world, with centers of diversity happening in Central and South America and Australia (Edmonds, 1978 ▶; D’Arcy, 1991 ▶)**.** It is locally known as *Tit Begun*, *Brihati Begun* (Bengali), *Pokhongkhesi* (Marma), *Titbahal* (Garo), etc. *Solanum violaceum* belongs to Solanaceae family which are extensively used as vegetables and fruits, but also used for various medicinal treatments. Traditionally it is useful in asthma, dry cough, catarrh, colic, flatulence, worms, and fever. Roots are digestive, carminative, and astringent to the bowels, cardiac tonic, expectorant, and aphrodisiac. Pounded root is worn in nasal ulcers and leaf juice mixed with ginger sap is used to stop vomiting. Fruits are used medicinally to relieve cough, alleviate toothache, and topically for skin disease. Fruits are considered anthelmintic, laxative, and digestive; helpful in pruritus, leucoderma. Seeds are mixed with ethnic liquor to increase intoxication effect and being used by the ‘Garo’ tribes in Bangladesh. The seeds are apparently used to extravagance gonorrhoea and dysuria (Jain and Borthakur, 1986 ▶). 

Proof of cytotoxic and anti-inflammatory activity of *S. violaceum *is found in some studies performed in some countries other than Bangladesh. The tests which have been carried out in the present study have not been conducted before on these plants native to Bangladesh, therefore, we chose the plant. In this study, our main goal was to evaluate possible chemical groups, total phenolic content (for assuming possible antioxidant potential), lethal effect on parasite (worms), and antimicrobial activity of *S. violaceum* to validate its use in folkloric treatments.

## Materials and Methods

**Plant materials collection**

For this research, *Solanum violaceum *was collected from, Noakhali, Bangladesh in July 2012 and was identified by Bangladesh National Herbarium, Mirpur, Dhaka (DACB Accession number: 37751).

**Chemicals**

Analytical grade chemical reagents used in the research were purchased from Merck, KGaA (Germany) and remaining are from BDH Laboratories (England) and UNI-CHEM (China).

**Preparation of plant materials**

The stems along with the leaves, fruits, and roots of the plant were cut into small pieces by a razor-sharp knife and dried in the sun (under a shadow) for five days. They were further dried in the oven at a temperature below 40 ^o^C for 24 h. About 1.5 kg of the dried plant materials was weighed by an electronic balance and grinded with a grinding machine.

**Extraction of plant materials**

Four hundred gram of the plant powder was macerated with 1500 ml of 99.8% methanol with sporadic shaking. After 12 days, the solvent was decanted and filtered using sterile cotton and Whatman^®^ filter paper No. 1 (Sargent-Welch, USA), and then evaporated at room temperature, and freeze-dried (yield 28 g deep greenish gummy extract).

**Phytochemical evaluation**

Small quantity of freshly prepared methanolic extract of *S. violaceum was* subjected to preliminary quantitative phytochemical analysis for the detection of phytochemicals such as alkaloids with Mayer’s and Hager’s reagent, Carbohydrates with Benedict’s test and Fehling’s test, glycosides with Legal’s test and Modified Borntrager’s test, phytosterols with Salkowski’s test and LibermannBurchard’s test, proteins with xanthoproteic test, flavonoids with alkaline reagent test and lead acetate test, tannins with gelatin test, saponins with Froth test and foam test, phenols with ferric chloride test, gums and mucilages** (**Harborne, 1993 ▶; Ansari, 2006 ▶; Dewan and Das, 2013 ▶; Roopashree, 2008 ▶). 

**Total phenolic content determination**

The amount of total phenolics in extracts was determined with the Folin-Ciocalteu reagent (Singleton and Rossi, 1965 ▶). Gallic acid was used as a standard and the total phenolics were expressed as mg/g of gallic acid equivalents (GAE). Concentration of 6.25, 12.5, 25, 50, and 100 µg/ml of gallic acid and concentration of 2 µg/ml of plant extract were also prepared in methanol and 0.5 ml of each sample were introduced into test tubes and mixed with 2.5 ml of a 10-fold dilute Folin-Ciocalteu reagent and 2 ml of 7.5% sodium carbonate. The tubes were covered with para-film and allowed to stand for 30 min at room temperature before the absorbance was read at 760 nm spectrophotometrically (UV-1800, Shimadzu, Japan). All determinations were performed in triplicate **(**Shahidi and Wanasundara, 1992 ▶). Total phenolic content was determined as mg of gallic acid equivalent per gram using the equation obtained from a standard gallic acid calibration curve. 

**Anthelmintic Activity**

The anthelmintic test was carried out with necessary modifications reported by Ajaiyeoba et al. (2001) ▶. The test was performed on adult earth worm (*Phertima posthuma*) because of its anatomical and physiological similarity with intestinal round worm parasite (Vidyarthi, 1967 ▶). The worms were collected from the moist soil at Noakhali, and identified by the Department of Fisheries and Marine Science (FIMS), Noakhali Science and Technology University (Voucher No. 18/2012). 

Methanolic extract of *S. violaceum* was used to prepare different concentrations (10, 20, 30, 40, and 50 mg/ml) of test sample separately. Fifteen mg of albendazole was dissolved in 10 ml water to prepare a concentration of 15 mg/ml. To confirm the validity of the test, a control group was established with distilled water. Earthworms were divided into six petri-dishes, each containing five earthworms. Five dishes were used for the five concentrations of methanol extract of *S. violaceum*. One group was applied to the reference standard and another for the control group. The time of paralysis was noted when no movement of any sort could be observed except when the worm was shaken vigorously. Time for death was recorded neither after ascertaining that worms moved neither when vigorously shaken nor when dipped in warm water (50 ˚C).

**Antibacterial and anti-fungal activity test**

*Test Organisms*

Two strains of Gram-positive (*Bacillus cereus, Bacillus subtilis*), three strains of Gram-negative bacteria (*Escherichia coli, Salmonella typhi, Vibrio cholerae*), and three strains of fungi (*Microsporum canis, Candida albicans, Aspergillus niger*) were used to evaluate the antimicrobial activity. The organisms were subcultured in nutrient broth and nutrient agar. They were collected from the Department of Microbiology, Chittagong Veterinary and Animal Sciences University, Bangladesh.

*Disc Diffusion Assay (DDA)*

Disc diffusion method is widely acceptable for the evaluation of antimicrobial activity (Bayer et al., 1966 ▶).

In this method, an antibiotic was diffused from a reliable source through the nutrient agar and a concentration gradient was created. Dried, sterilized filter paper discs (6 mm diameter, HI-Media, China) containing the known amounts of test samples (400 µg/disc) were placed on nutrient agar medium consistently seeded with the test bacteria. As positive and negative control, standard antibiotic of ciprofloxacin (5 µg/disc) and blank discs were used. For the maximum diffusion of the test materials to the surrounding media, these plates were reserved at low temperature (4 °C) for 24 h**.** The plates were then incubated at 37 °C for 24 h to allow optimum growth of the organisms. The test materials with antibacterial property inhibited microbial growth in plates and thereby yielded a clear, distinct zone defined as zone of inhibition. The activity of the test sample was then determined by measuring the zone of inhibition expressed in millimeter (Barry, 1976 ▶).

## Results

**Phytochemical screening**

Phytochemical study of the crude methanol extract of *S. violaceum *revealed the presence of alkaloid, carbohydrate, flavonoids, phenols, glycoside, saponin, gums, di-terpenes, proteins, and tannins (Table 1).

**Table 1:** Phytochemical screening of methanol extracts of *Solanum violaceum*

**Chemical Constituents**	**Test**	**Result**
Test for alkaloids	Mayer^’^s test	+
	Wagner^’^s test	+
	Hager^’^s test	+
Tests for carbohydrate	Molischs Test	+
Tests for reducing sugar	Benedict’s Test	-
	Fehling’s Test	-
Tests for Cardiac Glycoside	Legal’s test	+
Test for Flavonoids	Alkaline Reagent Test	+
	Lead acetate Test	+
Test for Saponins	Saponin test	+
Test for gums	Gum test	+
Test for phytosterols	Libermann-Burchard test	-
Test for tri-terpenes	Salkowski.s Test	-
Test for di-terpenes	Copper Acetate Test	+
Test for Phenols	Ferric Chloride Test	+
Test for Proteins	Xanthoproteic Test	+
Test for Tannins	Potassium dichromate Test	+

+ = presence, - = absence

**Total phenolic content determination**

Based on the absorbance values of the extract solutions, the colorimetric analysis of the total phenolics of the extract was determined and compared with that of the standard solution of gallic acid equivalents. Result (Table 2) shows the total phenolic amount calculated for *S*. *violaceum.* These results reported that total phenolic content of methanol extracts is correlated with the activity of gallic acid and showed that moderate amount of phenolics which play an important role in the antioxidant activity of plant materials.

**Effect on worms**

Time recorded for paralysis and death of earthworms for crude methanol extract of *S. violaceum* and standard drug are given in Table 3.

**Antimicrobial activity**

Antimicrobial activity of methanolic extract was tested against eight bacteria and fungi at concentrations of 400 μg/disc. Standard antibiotic disc of ciprofloxacin (5 μg/disc) was used for the comparison. The result of antimicrobial activity is given in Table 4. The extract revealed highest activity against *Aspergillus niger* (75%) and resistance to *V. cholera.*

The zone of inhibition of *S. violaceum* was very low; therefore, the MIC (minimum inhibitory concentration) was not determined.

**Table 2:** Total phenolic content determination of methanolic extract of *Solanum violaceum*

**Methanol extract**	**absorbance at 760 nm**	**Avg. absorbance at 760 nm ±SD**	**Total phenolic content of methanolic extract of ** ***Solanum violaceum***
Sample 01	0.324	0.325667±0.005	54.67±1.18 mg gallic acid equivalent (GAE) per gm of dry extract
Sample 02	0.331		
Sample 03	0.322

SD = Standard deviation

**Table 3:** Time for paralysis and death of earthworms for extract and standard

**Group**	**Concentration** **(mg/ml)**	**Paralysis time (min) Mean±SEM**	**Death time (min) Mean±SEM**
Sample 01	10	66.33**±** 3.05	81.67**±** 2.52
Sample 02	20	41.33**±** 2.08	53.67**±** 2.52
Sample 03	30	53**±**4	65**±**4
Sample 04	40	20.67**±** 1.53	30.67**±** 2.08
Sample 05	50	31.33**±** 2.52	39.67**±** 1.53
Standard	15	33.67**±** 1.53	51.33**±** 2.52

SEM = Standard error of mean, n = 5

**Table 4:** Antimicrobial activity of the crude sample of *Solanum violaceum*

**Test Organisms**		**Crude sample** **(400g/disc)**		**Ciprofloxacin** **(5 g/disc)**
		**Zone of inhibition**	**Relative % of inhibition**	
**Gram-positive Bacteria**	*Bacillus cereus*	11 mm	32%	34 mm
	*Bacillus subtilis*	14 mm	39%	36 mm
**Gram-Negative Bacteria**	*Escherichia coli*	3 mm	9%	32 mm
	*Salmonella typhi*	1 mm	5%	20 mm
	*Vibrio cholerae*	0 mm	0%	25 mm
**Fungi**	*Candida albicans*	2 cm	50%	4 cm
	*Aspergillus niger*	3 cm	75%	4 cm
	*Microsporum canis*	2 cm	66%	3 cm

## Discussion

Presence of different types of important phytoconstituents such as alkaloids, flavonoids, tannins, saponins, phenols, etc. reveals the usefulness of *S. violaceum* in various remedies. The methanolic extract of this plant revealed the presence of alkaloids, which may be used as analgesic antimicrobial, smooth muscle relaxant, anticencerous, antioxidant, etc. (Joshi et al., 2013 ▶). Flavonoids, which can be referred to as nature’s biological response modifiers, have shown anti-allergic, anti-inflammatory, antimicrobial, and anti-cancer activities and saponin can be used as mild detergents and in intracellular histochemical staining (Rievere et al., 2009 ▶). Studies tend to suggest that tannins may have significant value such as cytotoxic and antitumor (Joshi et al., 2013 ▶). The present study showed that phenolic compounds possess antidiabetic, antioxidative, and antimutagenic properties (Arts and Hollman, 2005 ▶). 

It has been recognized that the effects of antioxidant are mainly due to the phenolic compounds of the plant (Bandoniene et al., 2002 ▶). Polyphenolic compounds such as flavonoids, tannins, and phenols found in the plant extracts have been warranted to have multiple biological effects including antioxidant activity (Hasanuzzaman et al., 2013 ▶).

The study showed the presence of flavonoids and polyphenolic compound as one of the major chemical constituents responsible for anthelmintic activity (Paria et al., 2012 ▶). It is to be expected that tannins existed in the extracts of *S. violaceum* produced similar effects. Another potential anthelmintic mechanism of tannins is that they can bind to free protein in the GI tract of host animal on the cuticle of the parasite and cause death of parasite (Martin, 1985 ▶). 

Since the present study showed the presence of several bioactive secondary metabolites such as tannins, saponin, flavonoids, and alkaloids, that singly or in combination may be responsible for the defense mechanism against microorganisms and insects (Joshi et al., 2013 ▶). For the development of antimicrobial agents, plants are important resources of potentially useful structures, because they are available, thus cost-effective (Joshi et al., 2013 ▶); therefore *in vitro* antibacterial activity assay is the preliminary step towards this goal. 

In light of the results of the present study, it can be summarized that the plant extract possesses moderate antioxidant, broad-spectrum antimicrobial, and significant anthelmintic activities. Therefore, additional studies may be suggested to better understand the mechanism of such actions scientifically.
